# Nuclear localization of TEF3-1 promotes cell cycle progression and angiogenesis in cancer

**DOI:** 10.18632/oncotarget.7342

**Published:** 2016-02-12

**Authors:** Kaixuan Teng, Cuilan Deng, Jie Xu, Qiuxu Men, Tao Lei, Da Di, Ting Liu, Wenhua Li, Xin Liu

**Affiliations:** ^1^ Ministry of Education Laboratory of Combinatorial Biosynthesis and Drug Discovery, College of Pharmacy, Wuhan University, Wuhan, 430071, P.R. China; ^2^ College of Life Sciences, Wuhan University, Wuhan, 430072, P.R. China

**Keywords:** transcription enhancer factor 3 isoform 1, angiogenesis, cell cycle, HUVEC, cancer therapy

## Abstract

TEF3-1 (transcriptional enhancer factor 3 isoform 1), also known as TEAD4 (TEA domain family member 4), was recently revealed as an oncogenic character in cancer development. However, the underlying molecular pathogenic mechanisms remain undefined. In this paper, we investigated nuclear TEF3-1 could promote G1/S transition in HUVECs, and the expression levels of cyclins and CDKs were upregulated. Additionally, if TEF3-1 was knocked down, the expression of cyclins and CDKs was downregulated while the expression of P21, a negative regulator of the cell cycle, was upregulated. A microarray analysis also confirmed that TEF3-1 overexpression upregulates genes that are related to cell cycle progression and the promotion of angiogenesis. Moreover, we observed that nuclear TEF3-1 was highly expressed during the formation of vascular structures in gastric cancer (GC). Finally, tumor xenograft experiments indicated that, when TEF3-1 was knocked down, tumor growth and angiogenesis were also suppressed. Taken together, these results demonstrate for the first time that TEF3-1 localization to the nucleus stimulates the cell cycle progression in HUVECs and specifically contributes to tumor angiogenesis. Nuclear TEF3-1 in HUVECs may serve as an oncogenic biomarker, and the suppression of TEF3-1 may be a potential target in anti-tumor therapy.

## INTRODUCTION

TEF3-1 (transcriptional enhancer factor 3 isoform 1), also known as TEAD4 (TEA domain family member 4), belongs to a class of transcription factors, which are all widely expressed in eukaryotic cells. This transcription factor was found to bind to the MCAT motif of gene promoters [1, 2]. This gene product is a member of the transcriptional enhancer factor (TEF) family of transcription factors, which contain the TEA/ATTS DNA-binding domain. It is also called related transcriptional enhancer factor 1 (RTEF-1), transcription factor 13-like 1 (TCF13 L1), and related transcription enhancer factor 1B (hRTEF-1B). This gene is also critical for the establishment of the trophectoderm (TE)-specific transcriptional program in the preimplantation mouse embryo [3].

However, integrative genomics analyses have revealed the involvement of TEF3-1 in the multi-level dysregulation and oncogenic characteristics that are observed in gastric cancer (GC) and in the development of pancreatic cancer [4, 5]. In addition, the transcriptional coactivator Yes-associated protein (YAP) is a major regulator of organ size and proliferation, and increased YAP/TEF3-1 activity plays a causal role in cancer progression and metastasis [6–8]. As coactivators, YAP1 or TAZ interacts with the TEAD/TEF family of transcription factors (TEAD1-4) to transcriptionally regulate the target genes in tumor tissue [9–11]. Moreover, as a negative regulator of the YAP-TEAD transcriptional complex, VGL might be a new tumor suppressor target in the treatment of lung cancer [12, 13].

Tumor angiogenesis is critical for the formation of vascular structures and tumor metastasis. One of the hallmarks of tumor angiogenesis is the proliferation of endothelial cells, which is also regulated by cell cycle arrest. As a transcriptional stimulator of vascular endothelial growth factor (VEGF) in hypoxic endothelial cells, TEF3-1 induces an increase in angiogenic processes including the proliferation of endothelial cells and the formation of the vasculature [14–16]. This brought to our attention several recent clinical studies that have reported on the YAP/TEAD complex, which is activated in solid tumors such as liver, breast, and ovarian cancers [11, 17, 18]. Particularly, TEF3-1 is also found to be preferentially expressed in malignant mesothelioma (MM) and has been shown to promote the expression of specific genes that regulate cell proliferation and the cell cycle [19]. We presume that an understanding of the molecular mechanisms by which tumor angiogenesis is induced by TEF3-1 in endothelial cells.

Here, we show that nuclear TEF3-1 promoted G1/S cell cycle transition, but that a deficiency of TEF3-1 induced G1/S arrest and a decrease in the proliferation of HUVECs. A microarray analysis also indicated that TEF3-1 overexpression upregulated the expression of genes related to cancer, as most of the upregulated genes are involved in tumor angiogenesis. In addition, our data showed the presence of nuclear TEF3-1 protein in the tumor vasculature of gastric cancer, which suggests that the nuclear location of TEF3-1 in GC may be a biomarker for tumor progression and diagnosis. Finally, we observed that the knock down of TEF3-1 inhibited tumor angiogenesis in an animal model.

## RESULTS

### Overexpression of TEF3-1 and TEF3-1ΔNLS with a lentiviral vector in HUVECs

**Figure 1:**
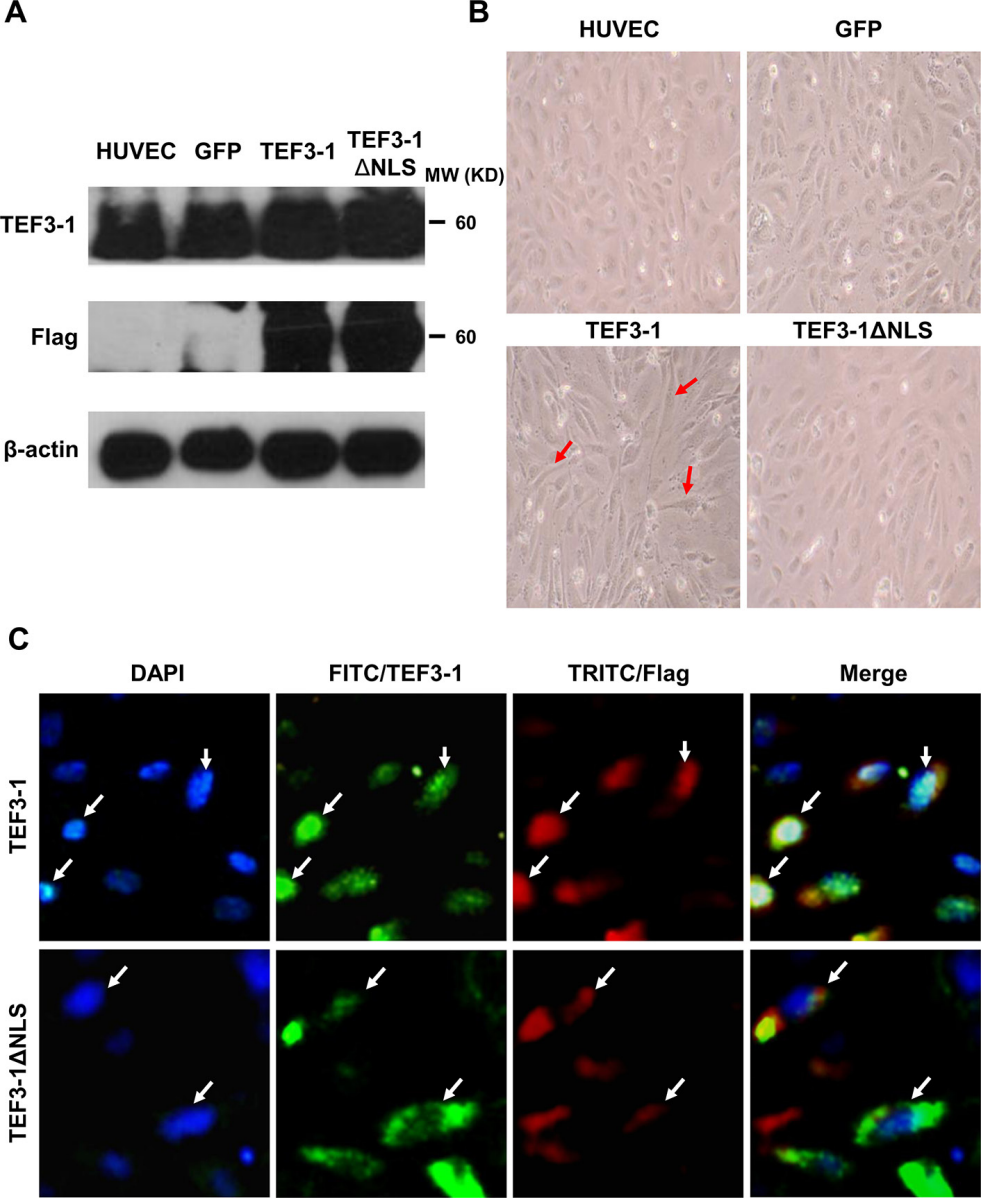
Expression of TEF3-1 or TEF3-1ΔNLS in HUVECs The mocked HUVECs were as the negative control. GFP was also expressed using lentiviral system to infect HUVECs as the other negative control. (**A**) Immunoblot analysis of the stable expression of the indicated proteins with Flag-tagged lentiviral vector in HUVECs. Full-length TEF3-1 or TEF3-1ΔNLS was detected using anti-TEF3-1 antibodies and anti-Flag antibodies. (**B**) Phase-contrast images of HUVECs with TEF3-1 or TEF3-1ΔNLS stable expression. Magnification × 200. (**C**) Immunofluorescence of HUVECs stained with Hoechst or labeled with anti-TEF3-1 antibodies or anti-Flag antibodies. Magnification × 200. The results shown are the sum of two independent experiments.

In previous experiments, the nuclear localization of TEF3-1 was first established by the induction of vascular endothelial growth factor (VEGF) and was found to contribute to proliferation, migration, tube formation, and angiogenesis [20]. However, the way in which TEF3-1 is regulated in tumor angiogenesis remains poorly understood. To explore the mechanism of TEF3-1 in HUVEC cells, this gene was overexpressed using a lentiviral system. After the HUVECs were infected with the Flag-tagged lentivirus, the protein levels of TEF3-1 and TEF3-1ΔNLS were examined by western blot analysis with antibodies against TEF3-1 or Flag; these antibodies recognize the tags that were fused with full-length TEF3-1 and TEF3-1ΔNLS, in which the nuclear location sequence (NLS) was deleted, respectively. This demonstrates that both TEF3-1 and TEF3-1ΔNLS were overexpressed in HUVECs, as expected (Figure 1A). Interestingly, TEF3-1 changed the morphology of HUVECs, which became more elongated than normal cells. Notably, when TEF3-1ΔNLS deleted the nuclear localization sequence, the mutant gene did not significantly change the morphology of the cells (Figure 1B). As shown in Figure 1C, the Flag-fused TEF3-1 was located in the nuclei, while the Flag-fused TEF3-1ΔNLS remained in the cytoplasm. This finding suggests that the function of this gene is associated with its cellular localization. To be consistent with these results, we also wanted to demonstrate the mechanism of the nuclear localization of TEF3-1 in HUVECs.

### TEF3-1 and its localization to the nucleus are required to promote G1/S transition

**Figure 2:**
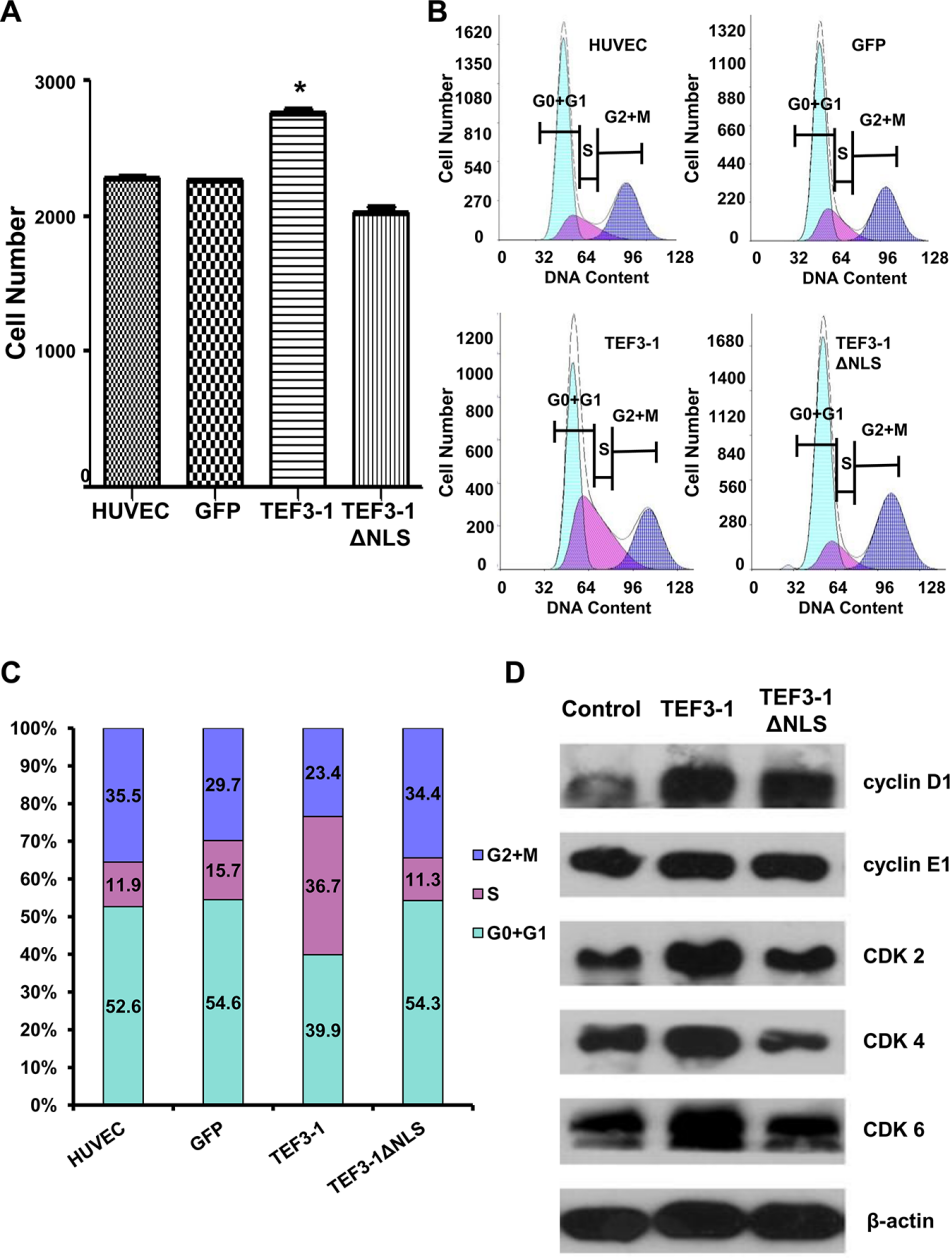
TEF3-1 and its localization to the nucleus affect the expression of G1/S cell cycle regulatory proteins in HUVECs The data represent an average of at least three independent experiments. The mocked HUVECs were as the negative control. GFP was also expressed using lentiviral system to infect HUVECs as the other negative control. (**A**) TEF3-1 increases the proliferation of HUVECs. HUVECs were treated with lentiviruses that express control vector, TEF3-1 and TEF3-1ΔNLS, respectively. The cell numbers were counted in a cell proliferation assay. *indicates *P* < 0.05 compared with the control. (**B** and **C**) The percentage of cells in each phase was determined by flow cytometric analysis. RNase and PI were added to the cell suspension after HUVECs were treated with lentiviruses that express GFP, TEF3-1 and TEF3-1ΔNLS, respectively. (**D**) Western blot assays were performed to detect the levels of cell cycle-related proteins. β-actin was used as the loading control.

First, to uncover how TEF3-1 expression would impact the cell cycle regulation of HUVECs, cell proliferation was observed after TEF3-1 overexpression. The stable expression of TEF3-1 was significantly increased in comparison with mock cells, while the expression of TEF3-1ΔNLS was not increased (Figure 2A). Furthermore, according to a flow cytometric analysis, we found that TEF3-1 expression promoted the transition of cell cycle from G1 to S phase and that the TEF3-1 nuclear localization was important for the promotion of cell cycle progress (Figure 2B and 2C). Additionally, a western blot analysis again demonstrated that TEF3-1 could increase the expression levels of cyclin E and CDKs, which are related to G1/S cell cycle transition. At the same time, TEF3-1ΔNLS expression did not clearly increase the expression levels of cyclin D and CDKs (Figure 2D). Together, these results indicate that TEF3-1 expression promotes G1/S transition in HUVECs and that the nuclear localization signal of TEF3-1 is also necessary for gene function. These results suggest a significant proliferative effect of the overexpression of TEF3-1 on cell cycle progress *in vitro*.

### Stably downregulated TEF3-1 expression suppresses cell proliferation and reduces the G1/S cell cycle signal *in vitro* in HUVECs

**Figure 3:**
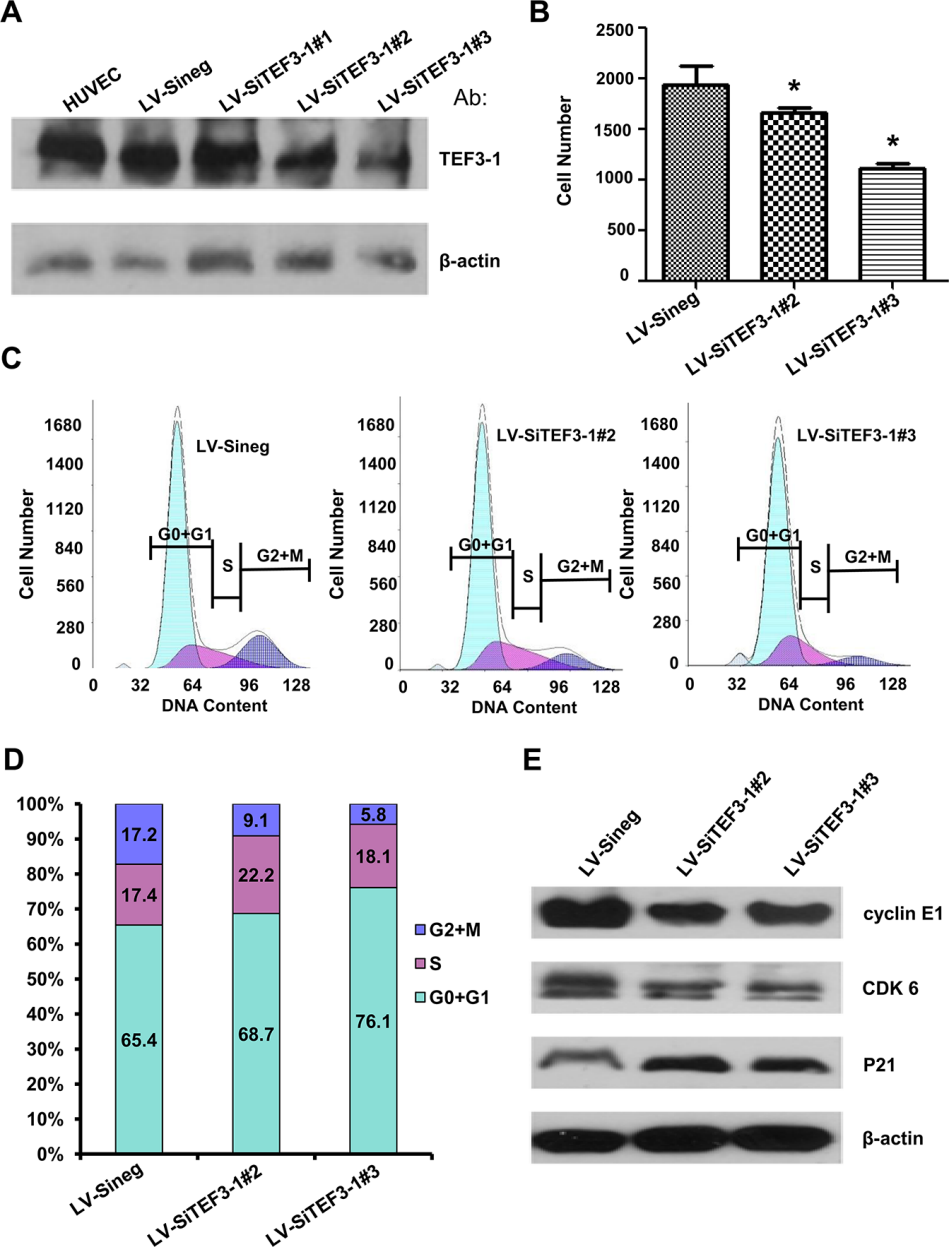
Depletion of TEF3-1 induces G1/S cell cycle arrest *in vitro* (**A**) Immunoblot analysis of TEF3-1 and actin expression in HUVECs infected with control lentivirus, LV-SiTEF3-1#2 and LV-SiTEF3-1#3, respectively.(**B**) HUVECs were treated with lentiviruses that express control vector and TEF3-1 SiRNAs, respectively. The cell numbers were counted in a cell proliferation assay. *indicates *P* < 0.05 compared with the control. (**C** and **D**) The percentage of cells in each phase was determined by flow cytometric analysis. RNase and PI were added to the cell suspension after HUVECs were treated with lentiviruses that express control, LV-SiTEF3-1#2 and LV-SiTEF3-1#3, respectively. (**E**) Immunoblot analysis of cell cycle-related proteins in HUVECs infected with control lentivirus, LV-SiTEF3-1#2 and LV-SiTEF3-1#3, respectively.

To demonstrate that the suppression of TEF3-1 expression could inhibit cell proliferation and G1 to S cell cycle transition, we used lentiviral-mediated SiRNA that specifically targets TEF3-1 to stably inhibit the expression of TEF3-1 in HUVECs. In this study, three types of lentiviral-mediated SiRNAs (LV-SiTEF3-1#1, LV-SiTEF3-1#2 and LV-SiTEF3-1#3) were used to suppress TEF3-1 protein in HUVECs and were compared with a mock vector. As shown in Figure 3A, LV-SiTEF3-1#2 and LV-SiTEF3-1#3 clearly downregulated TEF3-1 expression, especially LV-SiTEF3-1#3. Subsequently, we observed that cell proliferation ability was significantly reduced in comparison with the mock-treated cells (Figure 3B). Furthermore, we found that the knock down of TEF3-1 delayed G1 to S phase transition (Figure 3C and 3D). A western blot analysis demonstrated that the downregulation of TEF3-1 decreased the expression levels of cyclin E, CDKs and P21 (Figure 3E). These results also suggest an obvious effect of the downregulation of TEF3-1 on cell cycle progress in HUVECs.

### Microarray data indicates that TEF3-1 affects cell cycle- and angiogenesis-related gene expression in endothelial cells

**Figure 4:**
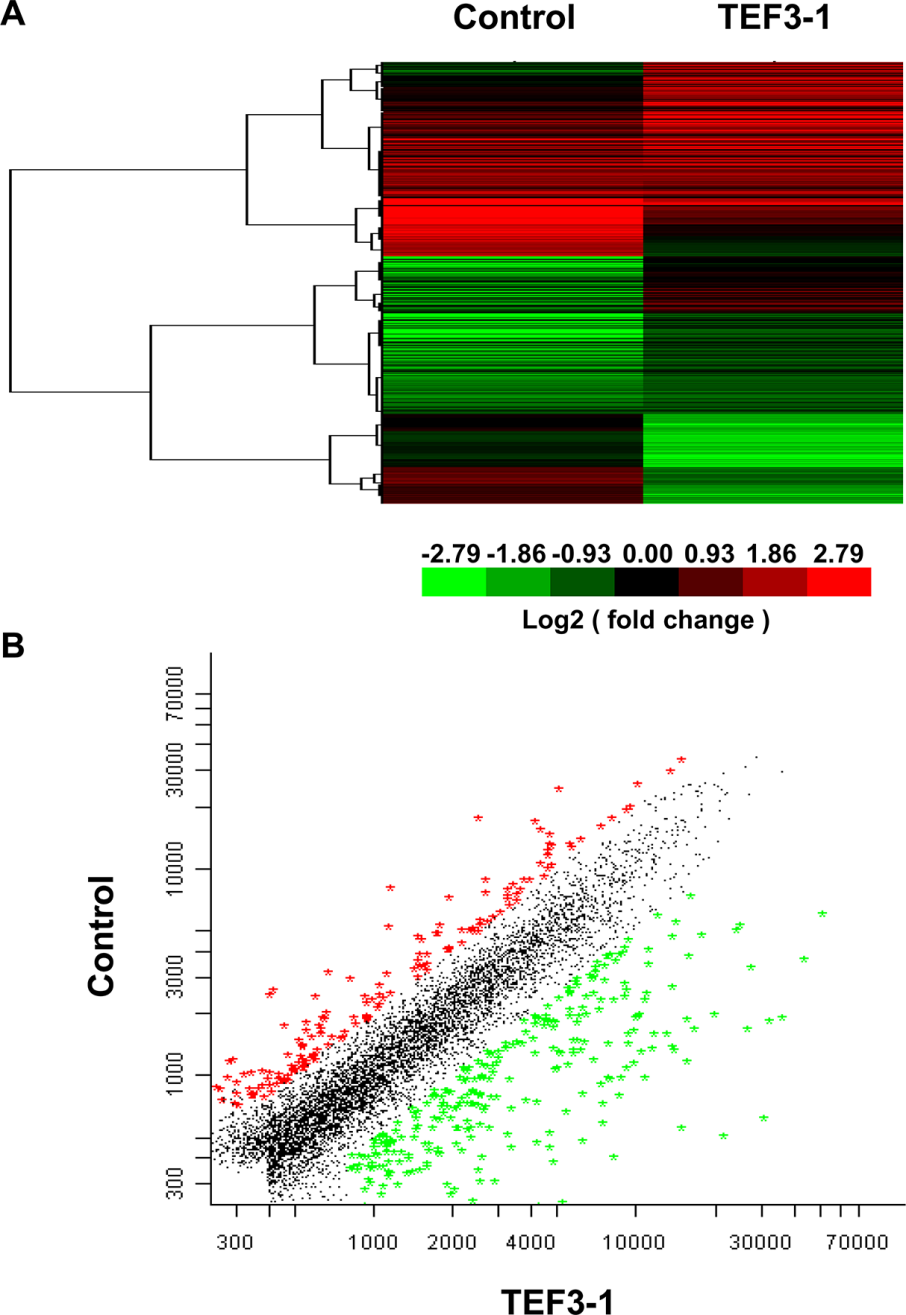

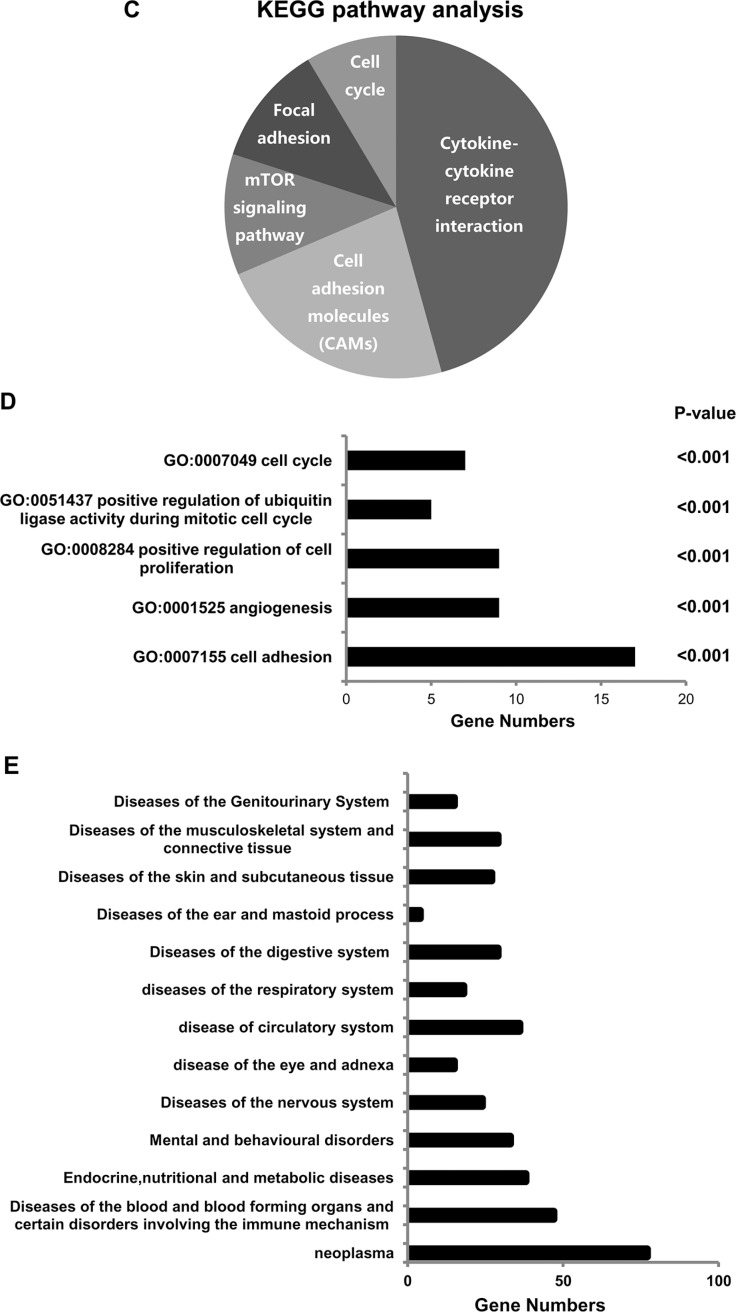
Microarray analysis reveals the functional importance of TEF3-1 in the cell cycle and in angiogenesis (**A**) Hierarchical clustering analysis of differentially expressed genes between LV-TEF3-1- and control lentivirus-infected HUVECs. Control lentivirus were packaged with empty plasmids. (Red) higher level of gene expression; (green) lower level of gene expression; (black) no change from the control. A color scale is shown below. (**B**) A scatter plot shows genes that are upregulated and downregulated compared with gene regulation observed with the control lentivirus. The different genes were selected based on a 2-fold (upregulated) or a 0.5-fold (downregulated) change threshold. (**C**) The KEGG pathway analysis shows that cell cycle regulation was one of the most frequently identified pathways. (**D**) Gene ontology of biological processes for differentially expressed genes using a molecular annotation system (MAS). Notably, these GOs are related to the cell cycle and angiogenesis with a *P*-value < 0.001 compared with control cells. (**E**) The different genes are involved in different diseases according to the ICD (International Classification of Disease) and 77 genes are involved in neoplastic diseases.

TEF3-1 was recently reported to possess functions in endothelial cells [15, 21], but additional mechanisms still need to be defined. In order to understand the mechanism by which TEF3-1 regulates cell cycle progression and angiogenesis in endothelial cells, we performed a microarray analysis. Then a 2-fold cut-off threshold for our microarray data were chosen to screen the different expressed genes. With the analysis of Cluster 3.0, Hierarchical clustering confirmed the different genes and their expression levels in the TEF3-1-infected HUVEC cells compared with the control-treated cells (Figure 4A). When we further analyzed the microarray results, we found that 108 genes were downregulated and 248 genes were upregulated (Figure 4B and Supplementary Table S1). Using these results and a KEGG pathway analysis [22], we explored signal pathways that may be regulated by TEF3-1 in HUVEC cells. As expected, one of the major pathways that was identified as the cell cycle-associated pathway (Figure 4C). To analyze signal pathways by GO analysis, the rank of cell cycle-associated pathways was considerably higher (Figure 4D and Supplementary Table S2). This suggests that one of the major functions of TEF3-1 in HUVECs is regulation of the cell cycle. In all, the genes including cyclins, cyclin-dependent kinases (CDKs), and cell division control (CDC) proteins, were directly associated with the cell cycle and were identified in the microarray.

Importantly, when TEF3-1 was overexpressed in HUVECs, angiogenesis was one of the important pathways in these cells. Moreover, compared with the other pathways, the rank of the adhesion pathway was the highest because it is one of the vital steps of tumor angiogenesis (Figure 4E). Importantly, when we sorted the different genes that are involved in different diseases according to the ICD (International Classification of Disease) and the disease database (http://www.malacards.org), we identified approximately 77 genes that are involved in neoplastic diseases (Figure 4E and Supplementary Table S3). These results strongly imply that TEF3-1 regulates the cell cycle in HUVECs and that this protein might play a role in tumor angiogenesis.

### Enhanced nuclear localization of TEF3-1 in endothelial cells in GC

**Figure 5:**
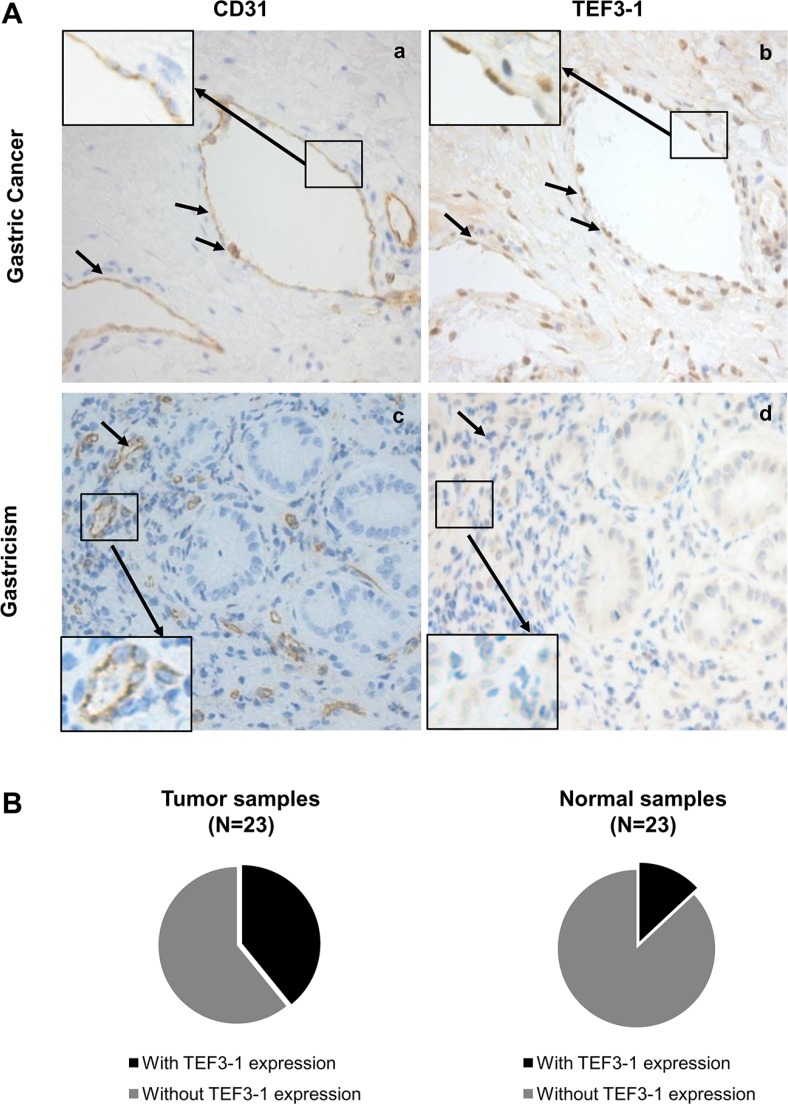
Frequent nuclear localization of TEF3-1 accompanied by CD31 expression in gastric cancer (**A**) Representative images from immunohistochemical staining for TEF3-1 and CD31 are presented for the GC and the matched normal tissues. 400x magnified figures are shown. The arrows indicate notable correlations between the expression of TEF3-1 and CD31. (**B**) Expression of TEF3-1 in 46 samples of GC and normal tissues.

Because nuclear TEF3-1 promotes the proliferation of endothelial cells, we wanted to confirm the specific localization of TEF3-1 in GC, thus, 46 samples were examined for TEF3-1 expression. Because CD31 is a marker of endothelial cells in tissue samples, an immunohistochemical analysis was performed with antibodies to CD31 and TEF3-1 in paired GC/normal tissues. In these tissues, we examined the protein expression levels and patterns of TEF3-1 expression in endothelial cells. We had 23 GC tumor samples available, and 9 out of 23 featured high expression of TEF3-1. According to IHC analysis, in 55% of the tumor tissues, TEF3-1 was located in the nuclei of both endothelial cells and tumor cells (Figure 5A, panels a and b). However, in the 23 matched normal tissues, only 3 out of 23 expressed TEF3-1, and in most cases, the expression was not in the nucleus (Figure 5A, panels c and d; *P* < 0.05).

In addition, the cancer tissues with a higher expression level of TEF3-1 also exhibited higher amounts of TEF3-1 in endothelial cells compared with normal tissues (Figure 5B). TEF3-1 was also found in most of the nuclei of GC tissues with TEF3-1 expression, which was consistent with a previous report that showed that TEF3-1 accumulates in the nuclei of HUVECs [20] (Supplementary Table S4). Therefore, TEF3-1 is indicated that was more frequently localized to the nucleus and accompanies angiogenesis in GC. Given that angiogenesis may be considered a required step for late-stage GC, TEF3-1 might be involved in the progression of GC.

### Knock down of TEF3-1 inhibits angiogenesis in Tumor Xenografts *in vivo*

**Figure 6:**
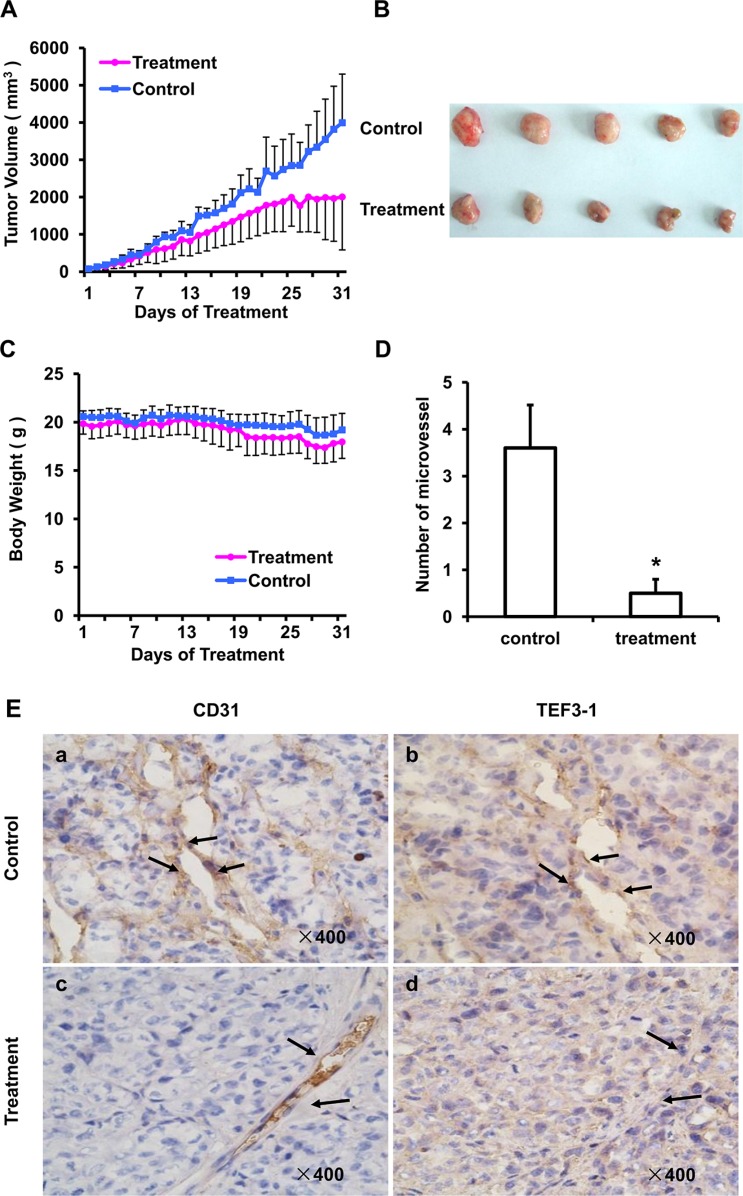
SiRNA of TEF3-1 inhibits angiogenesis in colon cancer *in vivo* HCT116 cells were inoculated into mice to establish a tumor model, as indicated in the materials and methods. Mice bearing tumors were randomly placed into two groups (5 mice/group) and were treated daily with LV-Sineg (control) or LV-SiTEF3-1#3 (treatment) for 31 days. The dose of injection was 1 × 10^6^ TU/ml, and animal weight and tumor volume were measured daily. (**A**) Mean tumor volumes after treatment. Values represent the means ± SD. *P < 0.05. (**B**) After 31 days of treatment, the *in vivo* tumors are shown in the images. (**C**) The mean values of the body weights of the mice. (**D**) Mean microvessel density of five fields that were randomly selected in each section after treatment; *n* = 5 for each group. Representative images of normal mouse tissues; values represent the means ± SD. (**E**) CD31 and TEF3-1 were evaluated by immunohistochemistry in tumor tissues derived from the SiRNA lentivirus-treated and the control-treated mice. Magnification: × 400.

As TEF3-1 can promotes endothelial cell proliferation, and the knock down of TEF3-1 inhibits G1/S cell cycle transition, we generated a colon cancer model to test whether LV-SiTEF3-1 could inhibit tumor vessel growth in nude mice. Nude mice bearing HCT116 tumors (approximate volume: 50 mm^3^) were randomly divided into two groups (5 mice/group) and were treated with LV-SiTEF3-1 or LV-Sineg control each day. The body weight of the mice and the tumor size were measured daily. As shown as Figure 6A, LV-SiTEF3-1 inhibited tumor growth. Treatment with LV-SiTEF3-1 also led to a slower increase in tumor weight, which is consistent with the increase in tumor volume (Figure 6B). Notably, we found no additional weight loss or other signs of toxicity, even in mice that were treated with LV-SiTEF3-1 for 31 days (Figure 6C).

Moreover, as vascular density could be measured in anti-CD31-stained vasculatures, the density of the vessels inside the tumors was observed in tumor sections after 31 days of treatment. The results of the immunohistochemical analysis showed a significant decrease in microvessel density in the LV-SiTEF3-1-treated group (Figure 6D), which further demonstrates the inhibition of vascular growth in colon cancer in nude mice. In order to evaluate the relationship between TEF3-1 expression and microvessel growth in the LV-SiTEF3-1-treated group and in the control group, an immunohistochemical analysis of mouse tumor tissues was performed using a specific anti-TEF3-1 antibody in the same field. As shown in Figure 6E, normal mouse tumors exhibited high levels of positive staining for both CD31 and TEF3-1 (Figure 6E, panels a and b). Notably, the majority of mouse tumors in the LV-SiTEF3-1-treated group expressed low levels of TEF3-1, even when they expressed CD31 (Figure 6E, panels c and d). Therefore, LV-SiTEF3-1 also exerts antitumor effects, and one of the important consequences is the inhibition of endothelial cell angiogenesis.

## DISCUSSION

Remarkably, we found that TEF3-1 plays roles in cell cycle regulation in endothelial cells, and a microarray analysis demonstrated that TEF3-1 overexpression promoted cell adhesion, cell cycle progression and angiogenesis. The inhibition of TEF3-1 was also confirmed in an animal model of colon cancer, where mice that were treated with the corresponding lentiviral vector demonstrated a significant reduction in tumor growth and vascular formation.

Previous studies have suggested that TEF3-1 is required for proliferation, migration, tube formation and angiogenesis of endothelial cells [14]. This may be because TEF3-1 is the transcription factor for VEGF, in addition to TEF3-1 can upregulate downstream genes such as DSCR1-1 L, EDG1, and FGF to induce angiogenesis in endothelial cells [15, 23–25]. Moreover, our data also indicate that VEGFC, VCAM and FOXC were upregulated with nuclear TEF3-1 overexpression in HUVECs, these genes are all involved in angiogenesis, promoting vascular and tumor growth (Supplementary Table S1) [26–28].

As an active transcription factor, TEF3-1 and its family members are also considered as part of the Hippo signaling pathway, which recently has been reported to be a pervasive oncogenic pathway [8, 29]. In this pathway, YAP (Yes-associated protein) promotes cell proliferation and angiogenesis and especially promotes tube formation of human microvascular endothelial cells in human cholangiocarcinoma via TEF3-1 transcription factors [30]. In pancreatic cancer, YAP1 also portends a novel mechanism for an oncogenic gene [5]. For the same reason that the YAP/TEAD complex is an important pathway in gastric cancer, VGLL4 and other peptides that specifically inhibit the YAP/TEAD complex are used to treat GC [4, 13, 31, 32]. Moreover, miRNAs that target the complex may also be developed as a promising therapeutic strategy [33]. For example, in one study, it was found that TEF3-1 interacted with KLF5 and suppressed p27 gene expression in triple negative breast cancers (TNBC) cell lines [34]. Therefore, TEF3-1 might also be a potential target and biomarker for the treatment of breast cancer.

Notably, although YAP was also recently reported to be an efficient anticancer target [5, 35, 36], it was noted that YAP could inhibit the growth of human malignant cells via apoptosis [37]. This indicated that YAP might have a dual function in human malignancies [38]. As a binding partner of YAP, TEF3-1 also exhibited functions both in the regulation of preimplantation mouse embryos [39] and in the regulation of malignant tumor cells [30, 40]. Usually, TEF3-1 and YAP family members are oncogenes [41], then the effect of nuclear location for TEF3-1 also can be cogitated in further study.

**Figure 7:**
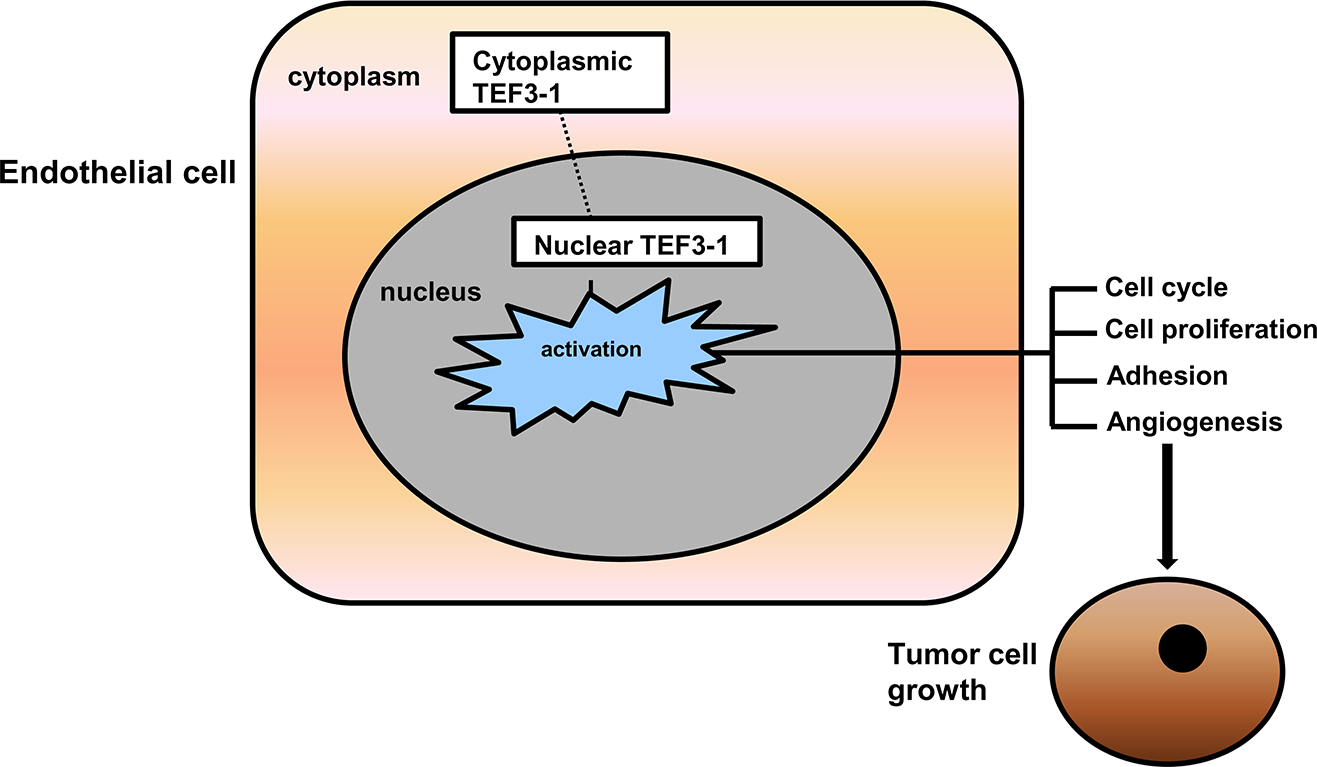
Putative model for the signaling pathway that mediates TEF3-1 induced angiogenesis It indicates nuclear TEF3-1 regulates HUVECs cell cycle, proliferation, adhesion and angiogenesis, thus promotes tumor growth.

Our results suggest that TEF3-1 overexpression is important for its accumulation in the nucleus. IHC experiments support the notion that TEF3-1 remains in the nucleus in GC tumor tissues, while normal tissues did not show the same location and expression patterns of TEF3-1. These results implied that the cellular distribution of TEF3-1 during tumor angiogenesis is regulated by nuclear retention. If TEF3-1 is expressed in the cytoplasm and not in the nucleus, then TEF3-1 cannot regulate tumor angiogenesis. If TEF3-1 was overexpressed and activated, nuclear TEF3-1 would then activate most of the downstream genes that induce cell cycle promotion and angiogenesis (Figure 7).

In summary, we have now unveiled a novel role for nuclear TEF3-1 in tumor angiogenesis and in the control of the cell cycle in endothelial cells. Thus, it would be very interesting to further explore the potential of using TEF3-1 nuclear expression as a mechanism-based prognostic biomarker or in combination with other potential markers in prognostic studies. Those data also suggest that the knock down of TEF3-1 may be an attractive antiangiogenic therapeutic target for the treatment of tumors.

## MATERIALS AND METHODS

### Ethics statement

Investigation has been conducted in accordance with the ethical standards and according to the Declaration of Helsinki and according to national and international guidelines and has been approved by the authors' institutional review board in College of Pharmacy, Wuhan University.

### Materials

EBM medium for HUVECs was purchased from Lonza (Walkersville, MD, USA). Antibodies against β-tubulin, cyclin D, cyclin E1, CDK2, CDK4, and CDK6 were obtained from Cell Signaling Technology (Boston, MA, USA). Experiments were repeated at least three times.

### Cell culture and lentiviral expression

HUVECs were cultured and transduced with lentiviruses that carry various genes, as previously described [16]. Briefly, the lentiviral vector was generated in HEK 293T cells by transfection with three plasmids. HEK 293T cells (5 × 10^6^) were transfected with 5 μg of the transfer plasmid, which encodes the expressed gene, 1.25 μg of the pMD2. G plasmid and 3.75 μg of the psPAX2 plasmid.

### Cloning and expression of TEF3-1 and SiRNAs

The TEF3-1 isoform was subjected to DNA sequence analysis. TEF3-1 and its mutations were subcloned into a pHAGE-Flag vector with primers BamHI-TEF3-1F (5′ CGCGGATCCTTGGAGGGCACGGCCGG 3′) and XholI-TEF3-1R (5′ CCGCTCGAGTCATTCTTTCA CCAGCCTGTAGA 3′). The TEF3-1ΔNLS contains the whole TEF3-1 cDNA without the CTGGCTCGTCGCAAA sequence, as described previously [20].

SiRNAs were designed using the website http://www.sirnawizard.com/design.php and were cloned into a pLKO.1 vector. The TEF3-1 SiRNA sequences were as follows: SiTEF3-1#1, GAGAATGGACACTACTCTTAC; SiTEF3-1#2, GACAGAGTATGCTCGCTATGA; SiTEF3-1#3, GCTGTGCATTGCCTATGTCTT.

### Cell proliferation assay

The effects of TEF3-1 overexpression on cell proliferation were characterized by cell counts. To assess cellular proliferation, a growth curve was generated after 0.5 × 10^6^ cells/well were seeded into a 6-well tissue culture plate. The cells were counted daily using a hemocytometer.

### Cell cycle analysis

After an incubation period of 22 h, various lentiviral vectors were added. HUVEC cells were harvested and treated with 70% ice-cold ethanol overnight. The cells were treated with RNase (50 μg/mL) at 37°C for 1 h and were then treated with PI (20 μg/mL) for 30 min at 4°C in the dark. The DNA content was analyzed by flow cytometry (Beckman Coulter).

### Western blot analysis

The cells were harvested and lysed in 1% SDS on ice. The supernatant was collected, and the protein concentration was determined with the Pierce BCA Protein Assay Kit (Thermo Scientific). Equivalent amounts of protein (20 μg) from each sample were transferred and incubated with the specific antibodies; the immunoblots were visualized by chemiluminescence. GAPDH was probed to ensure equal protein loading.

### Immunofluorescence

Briefly, cells were fixed in 4% paraformaldehyde, and the slides were blocked with 3% serum in phosphate buffered saline (PBS) with 0.1% Triton-X100. Cells were incubated with the primary antibodies overnight at 4°C and were then washed three times with 0.1% Tween-20 in PBS. Next, the slides were incubated with fluorescent-labeled secondary antibodies, and after several washes, the staining was visualized by fluorescence microscopy.

### Immunohistochemistry

Human tissue sections from the tissue microarray were deparaffinized, rehydrated through graded alcohol solutions. Antigen retrieval was performed by heating the sections in a 99°C water bath for 40 minutes. After endogenous peroxidase activity was quenched and nonspecific binding was blocked, the sections were incubated with antibodies to TEF3-1 (Abcam, Cambridge, MA) or CD31 (DAKO, Glostrup, Denmark) at room temperature for 30 minutes. Slides were incubated with the secondary antibody (Flex HRP) for 30 minutes. After the slides were washed, they were incubated with Flex DAB Chromogen and counterstained with Flex Hematoxylin.

### Microarray procedures

The cells were harvested and the total RNA was extracted. The commercially available 22 K Human Genome Array was purchased from CapitalBio Corporation (Beijing, China). Labeling, hybridization, washing and scanning were performed according to the standard operating procedure of CapitalBio Corporation. The obtained images were analyzed with LuxScan Version 3.0 (CapitalBio Corp.), which employed the LOWESS normalization method. The different expressed genes were analyzed using Molecular annotation system (MAS) and software Cluster 3.0 [42].

### Tumor xenografts

Five-week-old female BALB/c nude mice were obtained from the Animal Model Centre of Hunan Province (Changsha, Hunan, China). The HCT116 cells were implanted into the right flank of each mouse. When the tumor volume reached 50–100 mm^3^, the mice were randomly distributed into control and treatment groups (*n* = 5). The control group received the control lentiviral vector (LV-control) in PBS, whereas the treatment group was given LV-SiTEF3-1#3 for 31 days, after which the tumors were dissected, weighed and frozen.

### Microvessel staining analysis

As previously described, CD31 was used to stain microvessels in mouse tissue. Frozen sections were stained with rat anti-mCD31 antibody (BD Biosciences, Pharmingen) [43, 44], and the number of vessels was counted [45, 46].

### Statistical analysis

Data shown are presented as the means ± S.D. of at least three independent experiments. Statistical significance was considered at *P* < 0.05 according to Student's *t* test.

## SUPPLEMENTARY MATERIALS TABLES



## Abbreviations

TEF3-1: Transcription Enhancer Factor 3 Isoform 1
TEAD4: TEA Domain Family Member 4
YAP1: Yes-associated Protein 1
CDK: Cyclin-dependent Kinase
GC: Gastric Cancer
HUVEC: Human Umbilical Vein Endothelial Cells
HEK 293T cells: Human Embryonic Kidney 293T Cells
HCT116: Human Colon Cancer Cell 116
LV: Lentiviral Vector
NLS: Nuclear Localization Sequence
KEGG: Kyoto Encyclopedia of Genes and Genomes
GO: Gene Ontology
DSCR1-1L: Down Syndrome Candidate Region 1 Isoform 1
IHC: Immunohistochemistry
