# Supplementary material for: Nuclear localization of TEF3-1 promotes cell cycle progression and angiogenesis in cancer
Source: Oncotarget. 2016 Feb 12;7(12):13827–41. doi: 10.18632/oncotarget.7342 (PMC4924681; doi:10.18632/oncotarget.7342)
Supplement: Supplementary file 1 [file oncotarget-07-13827-s001.pdf]

Nuclear localization of TEF3-1 promotes cell cycle progression and angiogenesis in cancer

Supplementary Materials

Supplementary Table S1: The numbers of the upregulated and downregulated genes between the LV-TEF3-1 and the control lentivirus-infected HUVECs

248 up regulation genes

CCL23	HERC6	GCH1	TRIM14	SGTB	TPK1	ZDHHC14	ADAR
BF	RARRES3	C17orf27	HLA-G	APOL3	NP_115664	PANX1	NP_073594
ANGPTL1	PLEKHA4	PNPT1	GLRX	BST1	NP_116195	TAPBP	ELF1
CXCL10	ETV7	SLC40A1	TNFSF10	TRIM22	STXBP3	HTATIP2	NP_149988
TLR3	Q9H606	CXCL2	VEGFC	WNT7A	ERO1L	GALNT1	DNPEP
IFI30	CEACAM1	NP_954590	GP6	GPR2	HES4_ HUMAN	PDE4A	MMP7
MX2	UBE2L6	IL15RA	CCRL1	TLR4	XRN1	Y226_HUMAN	BCL6
LAMP3	C1orf29	FST	BTN3A3	BIRC3	NP_060840	NAGA	CNP
ECGF1	VCAM1	CFH	NT5C3	KE04_ HUMAN	NP_061060	BENE_ HUMAN	FOXC2
IFIT3	CCL5	STAT1	C1orf38	NMI	BTN3A2	MOV10	MMP1
LGALS3BP	NUP62	USP18	EXTL2	TNFAIP2	NAGK	P4HA1	TLE4
APOBEC3G	THSD2	ACSL5	MT1J	TOR1B	SCARB2	CMKOR1	ADM
Q96AL8	OAS3	B2M	NP_006691	NUB1_ HUMAN	C20orf18	TRIM38	LDHA
INDO	NP_060101	OASL	SLC25A28	PSME2	TNFRSF14	CD58	NP_775891
IF28_ HUMAN	LAP3	LGMN	NP_079232	NP_060765	TBC1D4	NP_116012	ABI3
BST2	NT5E	LOX	JAG1	TIMP1	NP_116258	NP_060388	TPST1
UBD	NP_077024	IRF7	PARP10	PPAP2B	MDK	ZNF183	CST3
IFITM1	CXCL3	SSA1	ZNF313	CD69	ADR2_ HUMAN	PLAUR	NP_071357
LGALS9	G1P3	TREX1	NP_060851	NP_443164	EVA1	SP22_HUMAN	VAMP8
IFI35	HLA-C	UBE1L	TNFAIP3	NP_056350	KIF13A	CTSL	EXOSC9
LY6E	MX1	CFHL2	NP_689755	CTRL	CCNA1	NFKBIA	NFE2L1
NP_612465	PLSCR1	SLC15A3	MAP3K5	SQRDL	DDHD1	SSBP2	HIVEP2
FAM46A	SAMHD1	WARS	CD47	NP_077027	B3GNT1	LIPA	OGFR
FBLN5	HERC5	TDRD7	MYD88	ANGPTL6	PSME1	BACH_ HUMAN	GARP

CX3CL1	EPSTI1	PSMB8	TAPBPL	PTPLA	GBP3	NP_851320	TXNDC
DDX58	IFIH1	ANGPTL4	SAT	MAP2	MPZL1	ITGA3	ADAM9
IFI44	GBP1	SOD2	PHF11	IFIT5	STARD4	P2RX4	DGKA
HAS3	POSTN	BAL_ HUMAN	ZC3HDC1	NP_005822	CCPG1	TCN2	RCN3
GBP4	PDGFRL	PNPT1	C13orf3	FVT1	IL6	COL8A1	ANTXR1
OAS1	CASP1	NCOA7	GOLPH2	HLA-E	ADAM10	SCARB2	GNPNAT1
MMRN1	NFE2L3	DUSP1	TRIM26	Y084_HUMAN	CD164	PSMA6	SCAMP2

108 down regulation genes

OPN3	TXNDC5	SLC7A7	NP_114149	BMP4	Q7Z4P3	TMEPAI
NP_057165	HSPA2	RPS9	GLTSCR2	DLC1	BCAT1	NP_112484
BTF3L2	C14orf31	RGS3	BOP1	PABPC4	DKK1	FREQ
NP_113653	RRM2	NP_057358	CDC20	ZNF235	SACS	JUB
EPS8L1	NP_653224	PLK2	VIM	C14orf151	LPXN	DLC1
PPP3CA	LXN	LIMK2	NMT2	EEF1G	KRT7	PGF
ANXA3	GFPT2	EIF4EBP1	NP_848591	CREB5	KIT	ANKRD1
FDPS	CCNA2	RPS3	Q9P2E8	MGLL	Q9H1N5	SERPIND1
DPH5_HUMAN	RPS7	RPL14	NP_620164	UACA	CERK	GPRC5B
C20orf108	NP_056244	CD34	NP_110446	EIF3S6IP	APLN	NP_443103
Q96DL5	CNN2	TOP2A	NP_078841	ITPKB	CAP2	NP_075059
BCAT1	RPL31	RPS5	NP_067650	NP_620141	EEF1A1	NP_054778
TTC3	C9orf19	SNCAIP	RPL34	RPL7	ESM1	TNFSF15
GPR116	ADAMTSL1	NP_443141	STMN1	PRKAR2A	FHOD1	GPR39
SIRT2	CDH2	A4GNT	RPL8	FABP5	EIF4B	PLAU
FABP4	C17_HUMAN	S100A2				

The regulated genes were selected based on 2-fold (upregulated) or 0.5-fold (downregulated) change threshold. Gene lists for 248 Up and 108 Down regulation genes with nuclear TEF3-1 overexpression in HUVECs.

Supplementary Table S2: The significant Gene ontology of biological process for different expressed genes using Molecular annotation system (MAS)

GO sample	gene numbers	*P*-value
GO:0007049 cell cycle	7	2.78E-05
GO:0051437 positive regulation of ubiquitin ligase activity during mitotic cell cycle	5	6.78E-09
GO:0008284 positive regulation of cell proliferation	9	2.78E-11
GO:0001525 angiogenesis	9	8.52E-14
GO:0007155 cell adhesion	17	6.23E-18

These GOs are related mostly to neoplasma with *P*-value < 0.001.

Supplementary Table S3: The different genes are involved in different diseases according to the ICD (International Classification of Disease)

The Name of Disease	gene number	gene name		
Neoplasma	77	up-regulate	59	ACSL5, ADAM10, ADM, ANGPTL4, ANTXR1, B2M, BST2, CASP1, CNP, LIPA, GARP, CNP, COL8A1, CCRL1, CXCL2, CXCL3, CCL5, DUSP1, ELF1, ECGF1, GCH1, GBP1, GP6, GLRX, HAS3, HERC5, HIVEP2, HLA-E, HLA-GVIM , HTATIP2, ITGA3, JAG1, LAP3, LGALS9, MAP2, MMRN1, MOV10, MT1J, NAGA, NAGK, NFKBIA, NPPC, OAS1, PANX1, PLSCR1, PNPT1, PPAP2B, RARRES3, SAT, SCARB2, SLC40A1, SOD2, TAPBP, TIMP1, TLR4, TNFAIP3, VCAM1, VEGFC, WNT7A
		down-regulate	18	ADAMTSL1, ANKRD1, BCAT1, CDC20, DKK1, EEF1G, FABP5, GPR39, PLAU, PLK2, PPP3CA, RPS7, RRM2, S100A2, SIRT2, VIM, TOP2A
Diseases of the blood and blood forming organs and certain disorders involving the immune mechanism	47	up-regulate	38	ADAR, ADM, B2M, BST1, CCL5, GARP, CNP, COL8A1, CCRL1, CXCL2, CXCL3, DUSP1, ELF1, GCH1, GP6, HERC5, HLA-E, HLA-G, JAG1, LAP3, MAP2, NAGA, NFE2L1, CNP, P4HA1, SAT, SCARB2, SLC40A1, TAPBP, TIMP1, TNFAIP3, TPK1, SSA1, VCAM1, VEGFC, WARS, BF, ECGF1, APOBEC3G, BST2
		down-regulate	9	ADAMTSL1, BMP4, CDC20, ESM1, FABP5, HSPA2, PLAU, PPP3CA, VIM
Endocrine, nutritional and metabolic diseases	38	up-regulate	32	ADAR, ADM, ANGPTL4, B2M, BST1, CASP1, CCL5, LIPA, CFH, GARP, CNP, DUSP1, HERC5, LAP3, LDHA, LIPA, GARP, MAP2, MMRN1, MOV10, NAGA, NFE2L1, CNP, PGF, SLC40A1, SOD2, TIMP1, TLR4, VCAM1, VEGFC, BF, ECGF1
		down-regulate	6	ADAMTSL1, ANKRD1, BMP4, CDC20, FABP4, PPP3CA
Mental and behavioral disorders	33	up-regulate	25	ADAR, ADM, APOL3, CEACAM1, LIPA, CFH, CTRL, CCRL1, DUSP1, HERC5, JAG1, LDHA, GARP, LY6E, MAP2, MOV10, NAGA, CNP, OAS1, PSMB8, PSME2, SOD2, BF, MX2, ECGF1
		down-regulate	8	ADAMTSL1, BMP4, CDH2, FABP5, FREQ, HSPA2, PLAU, S100A2

Diseases of the nervous system	24	up-regulate	21	ADAR, ADM, GBP3, CCL5, CNP, CXCL2, DUSP1, HERC5, LDHA, MAP2, MOV10, MT1J, NAGA, CNP, OAS1, P2RX4, PSME1, SOD2, VCAM1, MX2, BST2
		down-regulate	3	ADAMTSL1, PGF, S100A2
Disease of the eye and adnexa	15	up-regulate	12	ADM, CFH, GARP, CNP, CXCL2, HERC5, OAS1, PANX1, SOD2, TIMP1, VAMP8, VCAM1,
		down-regulate	3	ADAMTSL1, CERK, VIM
Disease of circulatory system	36	up-regulate	32	ADM, B2M, BST1, CCL5, LIPA, CFH, CNP, COL8A1, CCRL1, CXCL2, DUSP1ELF1, GP6, HIVEP2, ITGA3, JAG1, LAP3, LIPA, MAP2, MMRN1, MT1J, CNP, OAS1, PSMB8, SOD2, TIMP1, TLR4, VCAM1, VEGFC, WARS, ECGF1, BST2
		down-regulate	4	ADAMTSL1, ANKRD1, BMP4, PLAU,
Disease of the respiratory system	18	up-regulate	17	ADAR, ADM, CCL5, GARP, CNP, COL8A1, CTRL, CXCL2, MAP2, MMRN1, CNP, P4HA1, PHF11, SOD2, TLR4, VCAM1, ECGF1
		down-regulate	1	ADAMTSL1,
Disease of the digestive system	29	up-regulate	26	ADM, B2M, BST1, CCL5, LIPA, GARP, CNP, COL8A1, CXCL2, ELF1, HIVEP2, HLA-E, G1P3, JAG1, LAP3, LIPA, PSMB8, SLC40A1, SOD2, TAPBP, TIMP1, TIMP1, TLR4, VEGFC, BF, ECGF1
		down-regulate	3	CD34, DKK1, FABP5
Disease of the ear and mastoid process	4	up-regulate	4	CCL5, ITGA3, SOD2, TPK1
		down-regulate	0	
Disease of the skin and subcutaneous tissue	27	up-regulate	20	ANTXR1, B2M, CCL5, COL8A1, CXCL2, DUSP1, ITGA3, JAG1, MAP2, PHF11, PSMB8, RARRES3, SOD2, TIMP1, TNFAIP3, SSA1, VCAM1, WARS, BF, ECGF1
		down-regulate	7	ADAMTSL1, ADM, CDC20, FABP5, RPL7, S100A2, PLAU,
Disease of the musculoskeletal system and connective tissue	29	up-regulate	19	B2M, BST1, CCL5, COL8A1, CCRL1, CXCL2, ELF1, EXTL2, HAS3, HIVEP2, MT1J, PSMB8, SCARB2, SOD2, TAPBP, TIMP1, TLR4, ECGF1, BST2
		down-regulate	10	ADAMTSL1, BCAT1, CDC20, DKK1, FABP5, HSPA2, KRT7, PLAU, PPP3CA, RPL7,
Disease of the Genitourinary System	15	up-regulate	14	ADM, B2M, CCL5, CFH, COL8A1, CCRL1, LAP3, SOD2, TIMP1, TLR4, VCAM1, VEGFC, BF, BST2
		down-regulate	1	ADAMTSL1,

About proximately 77 genes were identified that are involved in neoplastic diseases.

Supplementary Table S4: Clinical information on 23 pairs of GC or Gastricism patients regarding age, gender, TEF3-1 expression and CD31 Correlation

CGC No.	Age	Gender	GC/Gastricism	STAGE	TEF3-1 EXPRESSION	CD31 CORRELATION
CGC-47	72	M	GC	IV	+	+
CGC-02	52	M	GC	II	–	–
CGC-03	79	M	GC	IV	+	+
CGC-04	61	M	GC	III	–	–
CGC-07	70	M	GC	III	+	–
CGC-08	63	F	GC	III	–	–
CGC-10	66	F	GC	III	–	–
CGC-11	52	M	GC	III	–	–
CGC-12	62	M	GC	III	+	+
CGC-16	69	M	GC	III	–	–
CGC-26	72	M	GC	III	–	–
CGC-39	57	M	GC	III	–	–
CGC-33	68	F	GC	II	–	–
CGC-34	63	M	GC	III	+	+
CGC-25	44	M	GC	III	–	–
CGC-37	63	M	GC	III	–	–
CGC-38	79	M	GC	III	–	–
CGC-30	74	M	GC	III	–	–
CGC-40	59	M	GC	II	–	–
CGC-36	62	M	GC	III	+	–
CGC-42	58	M	GC	II	+	–
CGC-46	68	F	GC	IV	+	+
CGC-05	41	M	Gastricism	×	–	–
CGC-06	48	M	Gastricism	×	–	–
CGC-09	34	M	Gastricism	×	–	–
CGC-13	47	M	Gastricism	×	–	–
CGC-14	45	M	Gastricism	×	–	–
CGC-15	24	M	Gastricism	×	–	–
CGC-17	74	M	Gastricism	×	–	–
CGC-18	36	M	Gastricism	×	–	–
CGC-19	57	M	Gastricism	×	–	–
CGC-20	47	M	Gastricism	×	–	–
CGC-35	62	M	Gastricism	×	–	–
CGC-32	41	M	Gastricism	×	–	–
CGC-21	52	M	Gastricism	×	+	–
CGC-22	47	M	Gastricism	×	–	–

CGC-23	78	M	Gastricism	×	+	–
CGC-24	48	M	Gastricism	×	–	–
CGC-27	25	F	Gastricism	×	–	–
CGC-28	51	M	Gastricism	×	–	–
CGC-29	58	M	Gastricism	×	+	–
CGC-31	66	M	Gastricism	×	–	–
CGC-71	30	F	Gastricism	×	–	–
CGC-75	45	M	Gastricism	×	–	–
CGC-78	63	M	Gastricism	×	–	–

'Positive correlation' indicates parallel patterns of expression levels or localization patterns between TEF3-1 and CD31. 'Independent' means that the patterns of expression between TEF3-1 and CD31 are not correlated.

M, male; F, female; GC, gastric cancer; +, positive; –, negative.
